# Risk of second primary thyroid cancer in cancer survivors

**DOI:** 10.1038/s41598-024-63155-z

**Published:** 2024-05-30

**Authors:** Yewei Yuan, Peng Sun, Hualin Xiao, Lingfan Li, Junyan Li, Xiang Ai

**Affiliations:** 1 Department of Breast Surgery, Sichuan Provincial Maternity and Child Health Care Hospital, No. 290 West Second Street, Shayan Road, Chengdu, 610031 Sichuan China; 2 Department of Thyroid and Breast Surgery, The General Hospital of Western Theater Command, Chengdu, 610083 Sichuan China; 3 Department of Thyroid and Breast Surgery, Chengdu Fifth People’s Hospital, The Fifth People’s Hospital Affiliated to Chengdu University of Traditional Chinese Medicine, Chengdu, 611130 Sichuan China

**Keywords:** Second primary thyroid cancer, Standardized incidence ratios, SEER database, Risk, Survival, Head and neck cancer, Cancer epidemiology

## Abstract

A risk factor for thyroid cancer (TC) may be a history of former cancer and cancer therapy. The precise risk of a second primary thyroid carcinoma has not yet been revealed. In this study, we evaluated standardized incidence ratios (SIRs) of second primary thyroid cancer (SPTC) with consideration of different conditions and further analyzed the clinicopathological characteristics and survival of these patients. The cohort was selected from the US Surveillance, Epidemiology, and End Results (SEER) Program between 1975 and 2019. The standardized incidence ratios, morbidity risk, clinicopathological features, and survival of second primary thyroid carcinoma were analyzed. Propensity score matching (PSM) was used to balance covariates. Kaplan–Meier method was performed to assess the survival outcomes. Overall, 7066 patients with SPTC and 83,113 patients with primary TC were identified. The SIR of TC in tumor patients was 1.51/10,000, statistically higher than the natural population (0.94/10,000, P < 0.05). The most significant tumors contributing to the increased SIRs of SPTC were acute lymphocytic leukemia (3.49/10,000), Hodgkin’s lymphoma-nodal (3.29/10,000), salivary gland cancer (3.23/10,000), and kidney and renal pelvis cancer (3.05/10,000). The incidence of TC increased significantly in tumor patients who received radiotherapy/chemotherapy before age 35. The age at diagnosis of the SPTC was much older than the primary TC (64.01 vs. 49.55 years, p < 0.001). The SPTC had a higher percentage of histological grades 3/4 (23.14% vs. 15.19%, p < 0.001). Survival analyses demonstrated a worse prognosis for the SPTC group compared to the primary TC group. But after PSM, the survival outcomes of the two groups tended to be equivalent (P = 0.584). The SIRs of TC are higher in tumor patients. The most significant factors contributing to the increased risk of SPTC were some specific former tumors and acceptance of radiotherapy/ chemotherapy before age 35. There was no significant difference in survival between SPTC and primary TC.

## Introduction

Thyroid cancer is one of the most common cancers. In recent years, the incidence of thyroid cancer has increased rapidly^1,2^. The causes of thyroid cancer and the increase in incidence are not well understood. Previous studies have suggested that a history of cancer and cancer treatment may be risk factors for thyroid cancer^3^.

The incidence of thyroid cancer has been reported to be rising among cancer survivors across the United States^4^. Data from a large cohort study showed an increased risk of TC in patients with primary breast cancer (BC) and vice versa^5,6^. The meta-analysis further confirmed that the ratio of secondary TC after BC was 1.55 (95% CI 1.44–1.67)^7^. In BC patients with CHEK2 mutations, the relative risk of thyroid cancer increased by three to nine times^8^. The positive association between thyroid cancer and other malignancies suggests that there may be a common genetic predisposition in some cases^9^.

Radiation therapy for primary cancer is the most recognized risk factor for secondary thyroid cancer after childhood cancer^10–12^, and studies have suggested that chemotherapy may also be associated with thyroid cancer^3,13^. However, other studies have shown that the increased risk of second primary thyroid cancer after malignancy does not appear to be due to treatment factors^4,14^.

Second primary thyroid cancer refers to a thyroid tumor found after a primary malignancy, excluding metastasis and recurrence. We evaluated the Standardized Incidence Ratios of second primary thyroid cancer after primary cancers using the SEER Program's population-based cancer registries in the United States, taking into account the site of the former tumor, time since the diagnosis of primary cancer, age at diagnosis, and initial treatment for the first primary malignancy. We then further analyzed the variables and survival of these second primary thyroid cancers.

## Materials and methods

### Data source

SEER*STAT software version 8.4.0 was used for obtaining SIRs and case listing for obtaining the study population for running propensity scoring based on the November 2021 submission (1975–2019 varying). This SEER program contains cancer incidence and mortality data from 8 population-based registries, representing approximately 8.3% of the US population (San Francisco-Oakland SMSA, Connecticut, Hawaii, Iowa, New Mexico, Seattle (Puget Sound), Utah, Atlanta (Metropolitan)).

### Selection and grouping of patients

We included primary TC patients and SPTC patients survived at least 12 months. SPTC patients were included if they were diagnosed with TC as their second cancer. We used the International Classification of Diseases for Oncology, 3rd edition histology and behavior codes (Site Recode ICD-O-3), and the World Health Organization 2008 criteria (WHO 2008) to identify TC patients and SPTC patients. We excluded cases diagnosed within six months after the primary tumors, cases with less than 20 SPTC observations (not present in Table 1), cases without radiation and chemotherapy information of former cancers, and cases with incomplete survival data and follow-up information. According to whether they had a history of a tumor before thyroid cancer, all patients were divided into two groups: the SPTC group and the primary TC group.

**Table 1:** Standardized incidence ratio of SPTC according to the site of former tumor.

Site of former tumor	Observe (no.)	SIR	CI lower	CI upper
Breast	2002	1.32*	1.26	1.38
Melanoma of the skin	745	1.57*	1.46	1.69
Prostate	704	1.21*	1.12	1.3
Corpus uteri	332	1.27*	1.13	1.41
Kidney and renal pelvis	306	3.05*	2.72	3.41
Lung and bronchus	274	2.10*	1.86	2.37
NHL—nodal	215	2.07*	1.8	2.36
Urinary bladder	171	1.11	0.95	1.28
Hodgkin—nodal	170	3.29*	2.81	3.82
Sigmoid colon	156	1.45*	1.23	1.7
Rectum	135	1.46*	1.22	1.73
Ovary	122	1.50*	1.24	1.79
NHL—extranodal	118	2.20*	1.82	2.63
Cervix uteri	100	0.95	0.77	1.16
Cecum	82	1.58*	1.26	1.96
Soft tissue including heart	80	2.76*	2.19	3.43
Miscellaneous	79	1.73*	1.37	2.15
Brain	69	2.39*	1.86	3.02
Testis	67	1.77*	1.37	2.25
Vulva	66	1.08	0.83	1.37
Stomach	63	2.40*	1.85	3.08
Larynx	61	2.06*	1.57	2.64
Chronic lymphocytic leukemia	57	1.40*	1.06	1.82
Rectosigmoid junction	51	1.38*	1.03	1.82
Tongue	50	2.17*	1.61	2.86
Ascending colon	49	1.23	0.91	1.63
Myeloma	48	1.77*	1.31	2.35
Salivary gland	46	3.23*	2.37	4.31
Small intestine	36	2.74*	1.92	3.8
Pancreas	36	2.54*	1.78	3.52
Other non-epithelial skin	33	1.54*	1.06	2.17
Acute lymphocytic leukemia	32	3.49*	2.39	4.93
Liver	31	2.69*	1.83	3.82
Gum and other mouth	30	2.25*	1.52	3.21
Descending colon	30	1.55*	1.05	2.22
Acute myeloid leukemia	29	2.65*	1.77	3.81
Transverse colon	28	1.26	0.84	1.82
Eye and orbit	27	2.31*	1.52	3.36
Bones and joints	23	2.15*	1.36	3.23
Anus, anal canal and anorectum	22	1.05	0.66	1.59
Other endocrine including thymus	21	3.78*	2.34	5.78

*SPTC* second primary thyroid cancer, *SIR* standardized incidence ratio, *CI* 95% confidence interval, *NHL* non-Hodgkin lymphoma.

*P < 0.05, compared with the natural population.

### Standardized incidence ratios of TC

Our main purpose was to analyze the impact of past cancers and their treatment on thyroid cancer incidence and prognosis. SIRs are calculated by dividing the number of observed cases of TC by the number of population-based expected counts. Rates were expressed per 100,000 people and adjusted for age using the 2000 U.S. standard population. A SIR > 1.0 can be interpreted as having more observed than expected events. SIRs of TC were calculated based on cancer and the natural population, respectively. For the SPTC, SIRs of different subgroups were counted according to gender (Male, Female), Race (White, Black, Other), behavior of the previous tumor (In situ, Malignant), age at diagnosis of previous tumor (15–44y, 45–54y, 55–64y, 65–74y, 75 + y), radiation of the previous tumor (Yes, No), chemotherapy of the previous tumor (Yes, No), and site of the previous tumor (Breast, Melanoma of the Skin, Prostate, etc.). The sites (No.) of carcinoma in situ were Breast (427), Melanoma of the Skin (229), Vulva (47), Sigmoid Colon (18), Corpus Uteri (14), Rectum (12), Anus, Anal Canal and Anorectum (5), Cecum (5), Larynx (5), Renal Pelvis (4), Descending Colon (2), Ascending Colon (2), Eye and Orbit (1), Pancreas (1), Prostate (1), Rectosigmoid Junction (1), Salivary Gland (1), Stomach (1), and Transverse Colon (1).

In order to explore the effect of radiotherapy and chemotherapy at different ages on the incidence of TC, SIRs were analyzed every five years according to age at diagnosis of the previous tumor.

### Variables and survival of TC

Independent demographic and clinicopathological variables of TC were compared between Group1 and Group2, including age, gender, year of diagnosis (1975–1990, 1991–2000, 2001–2010, and 2011–2019), histologic grade (grade 1, 2, 3, 4), histologic type (papillary follicular, papillary with follicular, medullary, and oxyphilic), T stage, N stage, M stage, surgery perform (yes, no), radiotherapy (yes, no), and chemotherapy (yes, no).

Thyroid cancer-specific survival (TCSS: The percentage of patients who have not died of TC, by NCI Dictionary of Cancer Terms) was calculated from the date of diagnosis to death from TC.

1:1 PSM (exact match, match tolerance = 0) was performed to further evaluate the effect of the previous tumor on TCSS by adjusting for age, gender, histologic grade, histologic type, T stage, N stage, M stage, surgery performed, radiotherapy, and chemotherapy.

### Statistical analysis

The excess risk of SIRs was calculated per 10,000. The statistical significance of SIRs between the SPTC and the primary TC was set at p < 0.05. SIR = standardized incidence rate of patients / standardized incidence rate of the general population to develop TC. SIR > 1 means patients have a higher risk of developing TC than the general population.

For demographic and clinicopathological data, continuous variables such as age were compared using the *t*-test or ANOVA test, and categorical variables were compared using Pearson’s chi-square test or rank sum test. Survival curves were performed according to the Kaplan–Meier method and compared using the log-rank test.

Multivariable Poisson regression models were used to compare SIRs among patient subgroups. Two-sided P-values for heterogeneity in SIRs were based on a likelihood ratio statistic comparing model. All confidence intervals (CI) were stated at the 95% confidence level. The statistical analyses were performed using SPSS (version 25.0, IBM Corp, Armonk, NY) and Stata statistical software (version 16.0, Stata Corp LLC, College Station, Texas).

### Compliance with ethics guidelines

This study was performed in accordance with the Helsinki Declaration of 1964, and its later amendments. This retrospective study was reviewed and approved by the Ethics Committee of The General Hospital of Western Theater Command. For this type of study formal consent is not required. No human participants or human tissue involved in this study.

## Results

From 1975 to 2019, a total of 91,071 patients with primary thyroid cancer were recorded. The SIR of thyroid cancer in the natural population was 0.94/10,000, with a prevalence of 1.36/10,000 in females, 0.5/10,000 in males, 0.37/10,000 before 35 years of age, and 1.49/10,000 after 35 years of age.

### SIRs of second primary thyroid cancer

7009 thyroid cancers occurred in patients with a history of other malignancies. The SIR of thyroid cancer in the tumor patient was 1.51/10,000, statistically higher than the natural population (P < 0.05).

The number and SIRs of the SPTC according to the site of the former tumor are shown in Table 1. Before the SPTC, breast cancer was the most common former malignancy, followed by melanoma and prostate cancer. The site of the previous tumor significantly affected the incidence of SPTC. The most significant tumors contributing to the increased SIRs of the SPTC were acute lymphocytic leukemia (3.49/10,000), Hodgkin’s lymphoma-nodal (3.29/10,000), salivary gland cancer (3.23/10,000), and kidney and renal pelvis cancer (3.05/10,000). The median time interval between the diagnosis of primary cancers and those of SPTCs and the percentage of patients who were diagnosed as SPTCs in the 1st year after primary cancer diagnosis with observed cases of more than 100 are shown in Supplement Table 1. 54.95% of patients with primary lung and bronchus tumors diagnosed as SPTCs in the 1st year (Supplementary Fig. 1). The median time interval between the diagnosis of Hodgkin’s lymphoma-nodal and SPTCs was 16 years.

The SIRs of thyroid cancer in different groups of tumor patients are shown in Table 2. In general, gender, race, in situ/malignant, radiotherapy, and chemotherapy of the former tumor had no significant effect on the incidence of SPTC. Although in most cases, age at diagnosis of the former tumor has little impact on the incidence, the incidence was significantly higher in patients who developed the first tumors before the age of 25 (3.14/10,000).

**Table 2:** Standardized incidence ratio of SPTC stratified by factors.

Variable		Observed (no.)	SIR	CI lower	CI upper
Total		7066	1.51*	1.48	1.55
Gender	Male	2384	1.64*	1.58	1.71
	Female	4682	1.46*	1.41	1.5
Race	White	5925	1.47*	1.43	1.5
	Black	308	1.65*	1.47	1.84
	Other	823	2.04*	1.90	2.19
	Unknown	10	0.27*	0.13	0.5
Behavior of former tumor	In situ	777	1.33*	1.24	1.43
	Malignant	6289	1.54*	1.5	1.58
Age at diagnosis of former tumor	0–24 y	293	3.14*	2.79	3.53
	25–34 y	413	1.64*	1.48	1.80
	35–44 y	897	1.42*	1.32	1.51
	45–54 y	1674	1.51*	1.44	1.59
	55–64 y	1857	1.48*	1.41	1.54
	65–74 y	1427	1.51*	1.43	1.59
	75 + y	505	1.35*	1.23	1.47
Radiation of former tumor	No	4881	1.50*	1.46	1.55
	Yes	1884	1.59*	1.52	1.66
Chemotherapy of former tumor	No	5288	1.46*	1.42	1.5
	Yes	1778	1.72*	1.64	1.8

*SPTC* second primary thyroid cancer, *SIR* standardized incidence ratio, *CI* 95% Confidence interval.

*P < 0.05, compared with the natural population.

**Figure 1:**
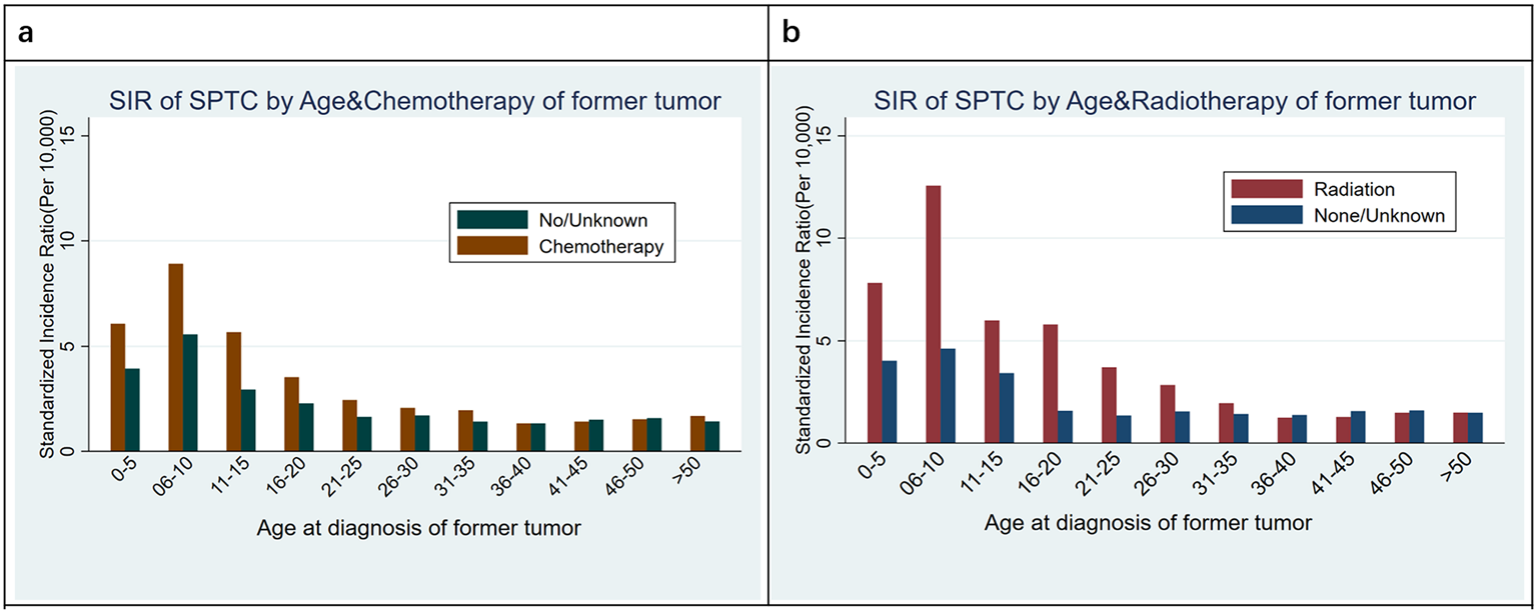
SIRs of second primary TC according to age at diagnosis and radiotherapy/chemotherapy of former tumor. (**a**) The incidence of TC increased in patients who received chemotherapy before the age of 35 years. (**b**) The incidence of TC increased in patients who received radiotherapy before the age of 35 years.

SIRs of the SPTC according to age at diagnosis and radiotherapy/chemotherapy of the former tumor are displayed in Fig. 1. The incidence of TC increased significantly in patients who received radiotherapy before the age of 35, but this effect was not evident after age 35. Similarly, chemotherapy in young patients aged 0–35 significantly increased the incidence of TC (Supplementary Tables 2 and 3). The most common former malignancies diagnosed before age 35 were Hodgkin’s lymphoma -Nodal, melanoma of the skin, breast cancer, brain tumor, and testis cancer (Supplementary Table 4).

### Comparison of demographics and clinicopathological characteristics between SPTC (Group1) and primary TC (Group2)

After excluding cases with incomplete information, there were 7,009 SPTC patients and 83,113 primary TC patients. For patients with SPTC, the average age at diagnosis of the former tumor was 54.02 years, and the mean interval between subsequent TC and the first tumor was 92.37 months.

The demographics and clinicopathological characteristics of the two groups are summarized in Table 3. The age at diagnosis of the SPTC was much older than the primary TC (64.01 vs. 49.55 years, p < 0.001). The proportion of males in the SPTC group was higher than that in the primary TC group (33.80% vs. 24.75%, p < 0.001). In terms of tumor characteristics, the distribution of pathological types was similar between the two groups. The SPTC had a higher percentage of histological grades 3/4 (23.14% vs. 15.19%, p < 0.001), and the proportion of T4 and M1 of the SPTC was slightly higher. As to treatment, a higher ratio of the SPTC did not complete surgery (11.88% vs. 5.44%, p < 0.001), and a lower proportion received radiotherapy (36.62% vs. 43.43%, p < 0.001).

**Table 3:** Demographics and clinicopathological characteristics.

Characteristics	No. of patients (%)		*P*
	Second primary TC (Group 1), n = 7009	Primary TC (Group 2), n = 83,113	
Age at diagnosis			
Mean ± SD, y	64.01 ± 14.23	49.55 ± 16.12	< 0.001
Gender			< 0.001
Female	4640 (66.20)	62,539 (75.25)	
Male	2369 (33.80)	20,574 (24.75)	
Race			
White	5878 (83.98)	66,838 (81.34)	< 0.001
Black	305 (4.36)	4057 (4.94)	
Other	816 (11.66)	11,280 (13.73)	
Unknown	10	938	
Year of diagnosis			< 0.001
1975–1990	397 (5.66)	12,820 (15.42)	
1991–2000	662 (9.44)	13,023 (15.67)	
2001–2010	2295 (32.74)	25,386 (30.54)	
2011–2019	3655 (52.15)	31,884 (38.36)	
Tumor grade			< 0.001
Grade I	810 (62.89)	10,607 (68.57)	
Grade II	180 (13.98)	2511 (16.23)	
Grade III	142 (11.02)	1090 (7.05)	
Grade IV	156 (12.11)	1260 (8.15)	
Unknown	5721	67,645	
Histologic type			< 0.001
Papillary	4390 (62.63)	52,785 (63.51)	
Follicular	417 (5.95)	5849 (7.04)	
Papillary with follicular	1492 (21.29)	18,039 (21.7)	
Medullary	135 (1.93)	1655 (1.99)	
Oxyphilic	206 (2.94)	1818 (2.19)	
Other	369 (5.26)	2967 (3.57)	
T stage			< 0.001
T0	19 (0.37)	83 (0.17)	
T1	3,160 (60.72)	28,777 (58.41)	
T2	743 (14.28)	8946 (18.16)	
T3	981 (18.85)	9431 (19.14)	
T4	301 (5.78)	2029 (4.12)	
Unknown	1805	33,847	
N stage			< 0.001
N0	3992 (76.78)	35,760 (73.38)	
N1	1207 (23.22)	12,973 (26.62)	
Unknown	1810	34,380	
M stage			< 0.001
M0	5284 (96.65)	49,483 (97.77)	
M1	183 (3.35)	1131 (2.23)	
Unknown	1542	32,499	
Surgery performed			< 0.001
Yes	6176 (88.12)	78,592 (94.56)	
No/unknown	833 (11.88)	4521 (5.44)	
Chemotherapy			0.021
Yes	99 (1.41)	922 (1.11)	
No/unknown	6910 (98.59)	82,191 (98.89)	
Radiotherapy			< 0.001
Yes	2567 (36.62)	36,095(43.43)	
No/unknown	4442 (63.38)	47,018(56.57)	
Died of TC			< 0.001
Yes	397 (5.66)	3704 (4.46)	
No/unknown	6612 (94.34)	79,409 (95.54)	

Anaplastic thyroid cancers were classified in the “other” histologic type subgroup.

*TC* thyroid cancer.

### Survival comparisons of SPTC (Group1) and primary TC (Group2)

The median duration of follow-up was 110 months for all TC patients. The 10-year and 20-year TC-specific mortality rates were 4.59% and 6.18%, respectively.

**Figure 2:**
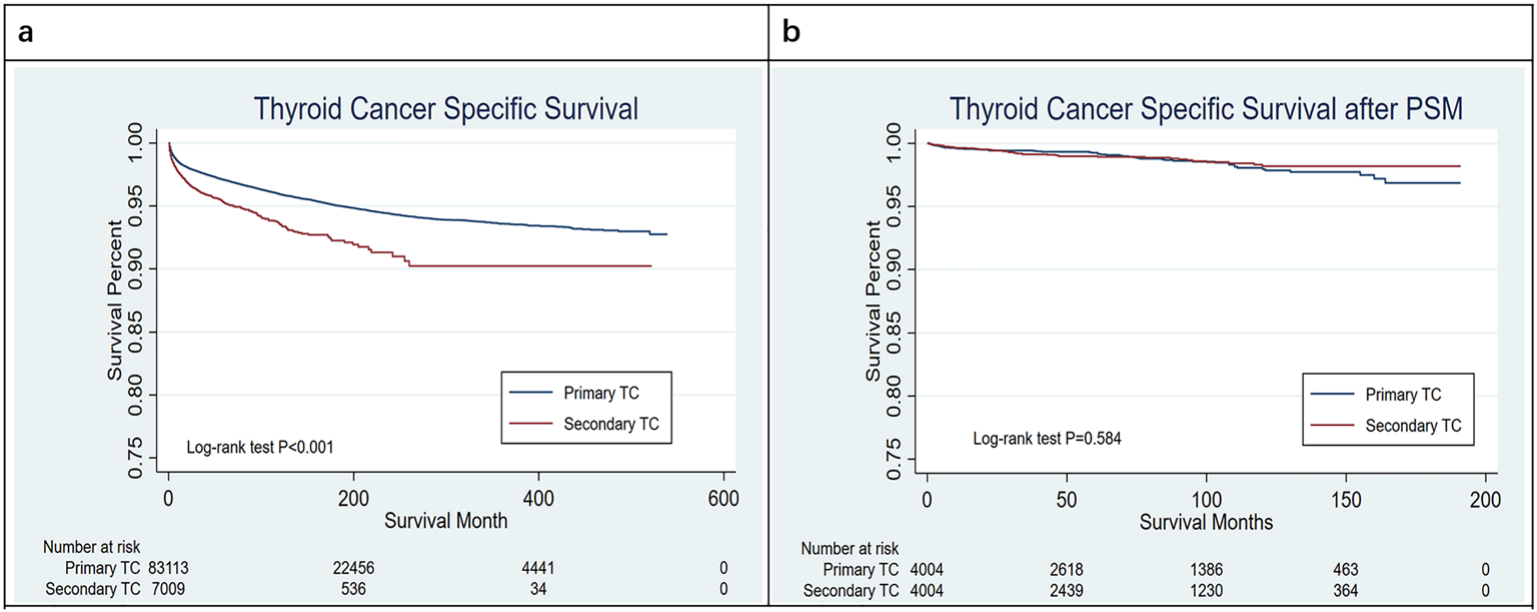
The analysis of thyroid cancer-specific survival curves of the primary TC and the secondary TC. (**a**) TCSS curves before PSM. (**b**) TCSS curves after PSM.

Kaplan–Meier analyses demonstrated a worse prognosis for the SPTC group (p < 0.001) (Fig. 2a). After 1:1 PSM, 4004 patients from each group were enrolled in the analysis (Supplementary Table 5). The survival outcomes of the two groups tended to be equivalent by adjusting for critical factors. (P = 0.584) (Fig. 2b).

## Discussion

This study indicated that malignant tumor survivors had a higher risk of developing thyroid cancer than the general population. The results were consistent with another study conducted by Schonfeld et al.^4^ The present study revealed a high risk for the development of SPTC after the diagnosis of acute lymphocytic leukemia, Hodgkin’s lymphoma-nodal, salivary gland cancer, and kidney and renal pelvis cancer. This was similar to the results of previous studies^4,15^. Despite the largely unknown molecular mechanisms and potential associations between primary tumors and subsequent thyroid malignancies, some progress has been achieved. Previous studies revealed that hormones, radiation exposure, and genetic susceptibility increased the risk for subsequent thyroid cancer among breast cancer survivors^7,16^. However, possible explanations for the potential increased risks of SPTC after lymphoid malignancies and other cancers are still unclear. Further investigation of these associations between primary malignancies and subsequent SPTC is warranted.

Malignant tumor survivors who received radiation therapy before the age of 35 significantly increased the incidence of thyroid cancer in this study. Previous studies have shown that radiation therapy can increase the incidence of well-differentiated thyroid cancer by 15–53 times^11,17^. Young people are at the highest risk after receiving radiation^12,18^. Taylor et al.^10^ indicated that childhood cancer survivors who received radiation therapy are more likely to develop thyroid cancer in the later stages than those tumor patients without radiation therapy. Patients with head-neck tumors, lymphomas, and ovarian cancers are regularly recommended for radiation therapy, and these patients have a significantly increased risk of developing thyroid cancer^19–21^. According to recent research by Polednik et al.^14^, radiation therapy for head-neck tumors does not increase the risk of developing thyroid cancer. This needs to be further confirmed by prospective studies.

Our study revealed for the first time that malignant tumor survivors who received chemotherapy before the age of 35 had a significantly increased incidence of thyroid cancer. Based on previous studies, it is still not certain if chemotherapy increases the risk of thyroid cancer. Veiga et al.^13^ showed an increased incidence of thyroid cancer in childhood cancer survivors who received chemotherapy containing alkylating agents. Hussein et al.^3^ revealed a significantly higher risk of SPTC in patients who received chemotherapy. Further research is warranted on detailed chemotherapy schemes for their effects on the incidence of thyroid cancer.

The latency between the first primary cancer diagnosis and the development of SPTC can guide clinicians in the formulation of appropriate treatment and surveillance strategies. At present, there are few relevant studies focused on latency trends. Lal et al.^22^ showed that many common cancers are associated with an increased risk of SPTC beyond 12 months of initial diagnosis. Schonfeld et al.^4^ revealed that SIRs of SPTC decreased after more than 5 years of primary cancer diagnosis, although this difference remained statistically significant. A recent study by Hussein et al.^3^ indicated different levels of risk for the development of SPTC after the diagnosis of different primary malignancies. However, they demonstrated that more than half of the SPTCs were diagnosed in the first three years after the primary cancer diagnosis. In sum, patients with primary cancer should be closely monitored for SPTC in the first 3 years, and the surveillance latency should be extended for some specific types of tumors, such as salivary gland, melanoma, stomach, lung, colon, ovarian, pancreas, prostate, and bladder.

This present study is the largest database to compare the demographics and clinicopathological characteristics of primary TC and SPTC. According to our results, from the perspective of epidemiology, the number of patients in both groups showed an increasing trend year by year, which may be associated with the improvement of modern medical detection methods as well as more regular physical examinations and postoperative surveillance. The proportion of females in the primary TC group was higher than that in the SPTC group (75.25% vs. 66.20%), and the average age in the primary TC group was significantly lower than that in the SPTC group (49.55 vs. 64.01). The pathological type of patients in both groups was mainly papillary carcinoma. The TNM stage was dominated by the early stage, the histological grade was dominated by grade I or grade II, and the proportion of patients receiving surgery (94.56% vs. 88.12%) and radiotherapy (43.43% vs. 36.62%) was higher in the primary TC group.

To our best knowledge, the current study is the first to compare the TCSS of primary TC and SPTC patients. Overall, SPTC patients had worse TCSS as compared to primary TC patients. Given the heterogeneous baseline characteristics, we performed a 1:1 PSM of the two groups. Survival analysis after PSM showed no statistical difference in TCSS between the two groups. According to other studies^2^, the mortality rates in children and adolescents with TC during 2008–2012 were less than 0.1 deaths per 1 million person-years, which means primary TC patients have a relatively good prognosis. The overall survival (OS) of patients in the two groups was not comparable due to the fact that the OS of patients with SPTC was greatly affected by the biological nature of the primary tumor. For this reason, we did not compare the OS for the two groups. Interestingly, Piek et al.^5^ showed that patients with BC-TC had better OS than patients with BC alone, and the underlying mechanism needs to be further investigated. There was no statistical difference in TCSS between the two groups, suggesting that for SPTC patients, both over- and under-treatment should be avoided. In the present study, the survival analysis of different primary tumor types of SPTC was not stratified, which will be further analyzed in subsequent studies.

Due to the retrospective nature of this study, missing information from the SEER database could not be verified. Data on the treatment of tumors are limited to information on radiation therapy and chemotherapy, while information on endocrine therapy and targeted therapy are unavailable. The main strength of the present study is that it represents a comprehensive investigation of the SIRs and the demographics and clinicopathological characteristics of primary TC and SPTC. Our research may contribute to the improvement of surveillance strategies. Further studies are warranted to illustrate the intrinsic mechanisms and associations between primary malignancies and SPTC.

## Conclusions

In conclusion, we found elevated TC risks following a variety of initial primary malignancies. Acceptance of radiotherapy/chemotherapy for the first primary malignancy before the age of 35 is related to an increased incidence of TC. Moreover, the survival analysis showed there was no significant difference in survival between SPTC and primary TC.

### Supplementary Information

Supplementary Figure 1.

Supplementary Tables.

## Data Availability

The datasets analysed in the current study are available from the corresponding author on reasonable request.
