# Supplementary figures and images for: Risk of second primary thyroid cancer in cancer survivors
Source: Sci Rep. 2024 May 30;14:12478. doi: 10.1038/s41598-024-63155-z (PMC11139851; doi:10.1038/s41598-024-63155-z)

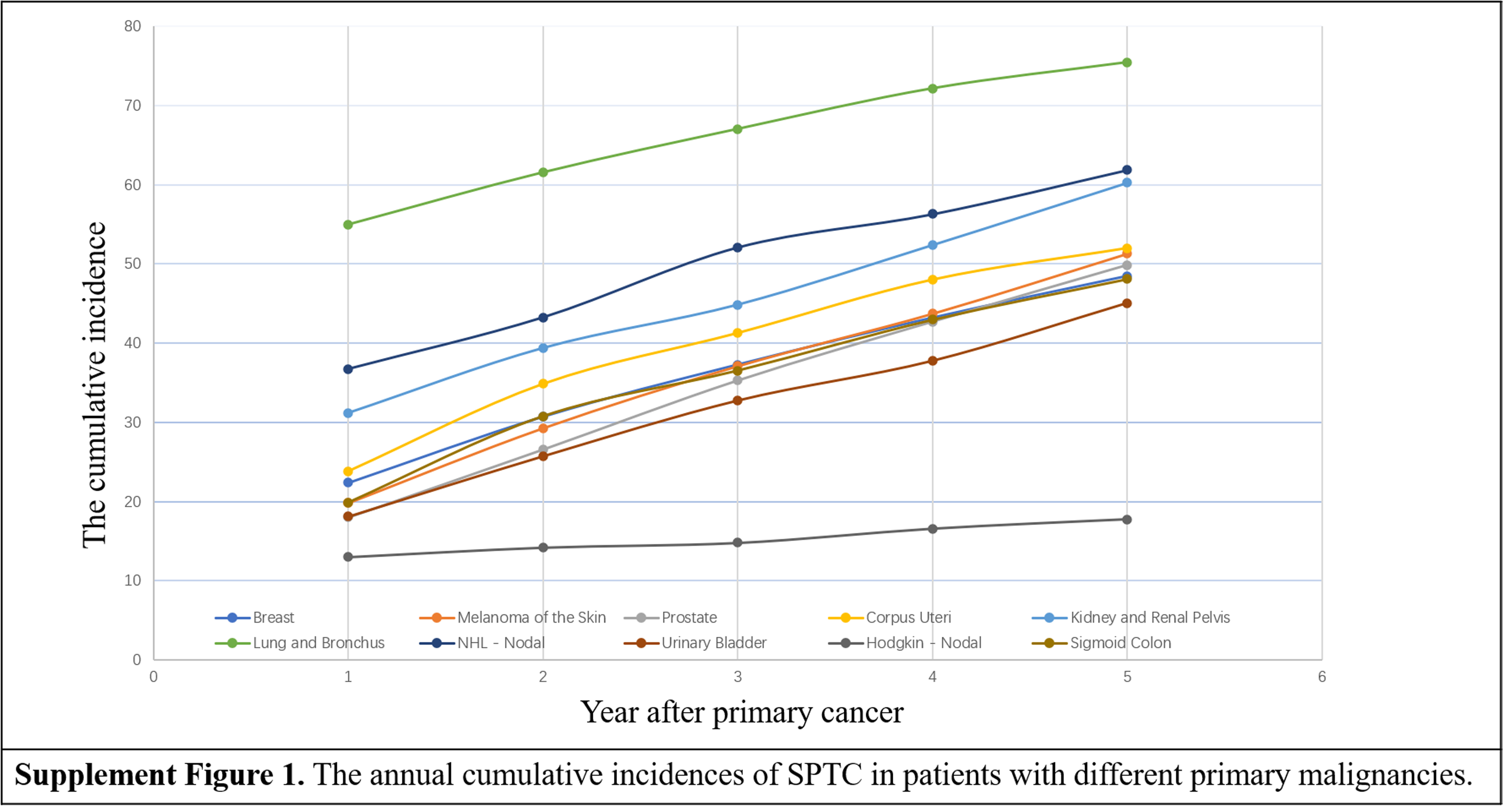

Supplement: Supplementary file 1 — Supplementary Figure 1. [file 41598_2024_63155_MOESM1_ESM.tif]
