# Supplementary material for: Risk of second primary thyroid cancer in cancer survivors
Source: Sci Rep. 2024 May 30;14:12478. doi: 10.1038/s41598-024-63155-z (PMC11139851; doi:10.1038/s41598-024-63155-z)
Supplement: Supplementary file 2 — Supplementary Tables. [file 41598_2024_63155_MOESM2_ESM.docx]

| **Supplementary Table 1.**  **The time interval between the diagnosis of primary cancers and SPTCs** | | | |
| --- | --- | --- | --- |
| **Site of former tumor** | **Observe (No.)** | **Median time interval (year)** | **Diagnosed in the 1st year (%)** |
| Breast | 2,002 | 6 | 22.40 |
| Melanoma of the Skin | 745 | 5 | 19.84 |
| Prostate | 704 | 6 | 18.00 |
| Corpus Uteri | 332 | 5 | 23.85 |
| Kidney and Renal Pelvis | 306 | 4 | 31.16 |
| Lung and Bronchus | 274 | 1 | 54.95 |
| NHL - Nodal | 215 | 3 | 36.74 |
| Urinary Bladder | 171 | 7 | 18.13 |
| Hodgkin - Nodal | 170 | 16 | 13.02 |
| Sigmoid Colon | 156 | 6 | 19.87 |
| Rectum | 135 | 5 | 23.13 |
| Ovary | 122 | 7 | 24.79 |
| NHL - Extranodal | 118 | 2 | 46.96 |
| Cervix Uteri | 100 | 5 | 35.00 |
| **Abbreviations**: SPTC, Second primary thyroid cancer; NHL, non-Hodgkin lymphoma | | | |

| **Supplementary Table 2.**  **SIR of SPTC according to age at diagnosis and radiotherapy of former tumor** | | | | | |
| --- | --- | --- | --- | --- | --- |
| **Age at diagnosis** | **Radiotherapy** | **Observed (No.)** | **SIR** | **CI Lower** | **CI Upper** |
| 0-5 | None/Unknown | 23 | 4.02* | 2.55 | 6.04 |
|  | Beam radiation | 23 | 7.81* | 4.95 | 11.72 |
| 6-10 | None/Unknown | 17 | 4.62* | 2.69 | 7.4 |
|  | Beam radiation | 29 | 12.56* | 8.41 | 18.03 |
| 11-15 | None/Unknown | 25 | 3.42* | 2.21 | 5.05 |
|  | Beam radiation | 22 | 6.00* | 3.76 | 9.08 |
| 16-20 | None/Unknown | 30 | 1.58* | 1.06 | 2.25 |
|  | Beam radiation | 41 | 5.80* | 4.16 | 7.87 |
| 21-25 | None/Unknown | 58 | 1.34* | 1.02 | 1.73 |
|  | Beam radiation | 43 | 3.71* | 2.69 | 5 |
| 26-30 | None/Unknown | 129 | 1.54* | 1.28 | 1.83 |
|  | Beam radiation | 58 | 2.84* | 2.15 | 3.67 |
| 31-35 | None/Unknown | 188 | 1.42* | 1.23 | 1.64 |
|  | Beam radiation | 70 | 1.95* | 1.52 | 2.47 |
| 36-40 | None/Unknown | 270 | 1.37* | 1.21 | 1.54 |
|  | Beam radiation | 85 | 1.24 | 0.99 | 1.53 |
| 41-45 | None/Unknown | 436 | 1.56* | 1.42 | 1.71 |
|  | Beam radiation | 147 | 1.27* | 1.08 | 1.5 |
| 46-50 | None/Unknown | 573 | 1.59* | 1.46 | 1.73 |
|  | Beam radiation | 231 | 1.48* | 1.3 | 1.69 |
| >50 | None/Unknown | 3,132 | 1.48* | 1.43 | 1.53 |
|  | Beam radiation | 1,135 | 1.49* | 1.4 | 1.57 |
| **Abbreviations**: SIR, Standardized incidence ratio; SPTC, Second primary thyroid cancer; CI, 95% Confidence interval; *, P < 0.05 | | | | | |

| **Supplementary Table 3.**  **SIR of SPTC according to age at diagnosis and chemotherapy of former tumor** | | | | | |
| --- | --- | --- | --- | --- | --- |
| **Age at diagnosis** | **Chemotherapy** | **Observed (No.)** | **SIR** | **CI Lower** | **CI Upper** |
| 0-5 | No/Unknown | 11 | 3.93* | 1.96 | 7.03 |
|  | Yes | 36 | 6.07* | 4.25 | 8.4 |
| 6-10 | No/Unknown | 13 | 5.57* | 2.96 | 9.52 |
|  | Yes | 33 | 8.91* | 6.14 | 12.52 |
| 11-15 | No/Unknown | 16 | 2.94* | 1.68 | 4.78 |
|  | Yes | 32 | 5.68* | 3.89 | 8.02 |
| 16-20 | No/Unknown | 39 | 2.28* | 1.62 | 3.12 |
|  | Yes | 33 | 3.53* | 2.43 | 4.96 |
| 21-25 | No/Unknown | 67 | 1.64* | 1.27 | 2.08 |
|  | Yes | 36 | 2.44* | 1.71 | 3.38 |
| 26-30 | No/Unknown | 140 | 1.70* | 1.43 | 2.01 |
|  | Yes | 51 | 2.06* | 1.54 | 2.71 |
| 31-35 | No/Unknown | 179 | 1.41* | 1.21 | 1.63 |
|  | Yes | 90 | 1.95* | 1.57 | 2.4 |
| 36-40 | No/Unknown | 258 | 1.33* | 1.17 | 1.5 |
|  | Yes | 108 | 1.33* | 1.09 | 1.61 |
| 41-45 | No/Unknown | 430 | 1.50* | 1.36 | 1.64 |
|  | Yes | 172 | 1.41* | 1.21 | 1.64 |
| 46-50 | No/Unknown | 604 | 1.58* | 1.46 | 1.71 |
|  | Yes | 232 | 1.52* | 1.33 | 1.72 |
| >50 | No/Unknown | 3,531 | 1.42* | 1.37 | 1.47 |
|  | Yes | 955 | 1.68* | 1.58 | 1.79 |
| **Abbreviations**: SIR, Standardized incidence ratio; SPTC, Second primary thyroid cancer; CI, 95% Confidence interval; *, P < 0.05 | | | | | |

| **Supplementary Table 4. Former tumor site of patients ≤35 years old** | | | |
| --- | --- | --- | --- |
| **Site of former tumor** | **Freq（No.）** | **Percentage（%）** | **Cumulation（%）** |
| Hodgkin - Nodal | 140 | 18.18 | 18.18 |
| Melanoma of the Skin | 121 | 15.71 | 33.9 |
| Breast | 75 | 9.74 | 43.64 |
| Brain | 49 | 6.36 | 50 |
| Testis | 40 | 5.19 | 55.19 |
| NHL - Nodal | 38 | 4.94 | 60.13 |
| Acute Lymphocytic Leukemia | 31 | 4.03 | 64.16 |
| Cervix Uteri | 26 | 3.38 | 67.53 |
| Ovary | 24 | 3.12 | 70.65 |
| Corpus Uteri | 17 | 2.21 | 72.86 |
| Kidney | 17 | 2.21 | 75.06 |
| Soft Tissue including Heart | 17 | 2.21 | 77.27 |
| Salivary Gland | 16 | 2.08 | 79.35 |
| Acute Myeloid Leukemia | 15 | 1.95 | 81.3 |
| Bones and Joints | 14 | 1.82 | 83.12 |
| NHL - Extranodal | 14 | 1.82 | 84.94 |
| Vulva | 12 | 1.56 | 86.49 |
| Lung and Bronchus | 9 | 1.17 | 87.66 |
| Other Non-Epithelial Skin | 8 | 1.04 | 88.7 |
| Miscellaneous | 7 | 0.91 | 89.61 |
| Cranial Nerves Other Nervous System | 6 | 0.78 | 90.39 |
| Adrenal Gland | 4 | 0.52 | 90.91 |
| Eye and Orbit - Melanoma | 4 | 0.52 | 91.43 |
| Hepatic Flexure | 4 | 0.52 | 91.95 |
| Myeloma | 4 | 0.52 | 92.47 |
| Rectum | 4 | 0.52 | 92.99 |
| Eye and Orbit - Non-Melanoma | 3 | 0.39 | 93.38 |
| Larynx | 3 | 0.39 | 93.77 |
| Liver | 3 | 0.39 | 94.16 |
| Nasopharynx | 3 | 0.39 | 94.55 |
| Pancreas | 3 | 0.39 | 94.94 |
| Sigmoid Colon | 3 | 0.39 | 95.32 |
| Cecum | 2 | 0.26 | 95.58 |
| Chronic Myeloid Leukemia | 2 | 0.26 | 95.84 |
| Descending Colon | 2 | 0.26 | 96.1 |
| Gum and Other Mouth | 2 | 0.26 | 96.36 |
| Lip | 2 | 0.26 | 96.62 |
| Mediastinum and Other Respiratory Org | 2 | 0.26 | 96.88 |
| Other Endocrine | 2 | 0.26 | 97.14 |
| Other Female Genital Organs | 2 | 0.26 | 97.4 |
| Other Lymphocytic Leukemia | 2 | 0.26 | 97.66 |
| Urinary Bladder | 2 | 0.26 | 97.92 |
| Acute Monocytic Leukemia | 1 | 0.13 | 98.05 |
| Anus, Anal Canal and Anorectum | 1 | 0.13 | 98.18 |
| Chronic Lymphocytic Leukemia | 1 | 0.13 | 98.31 |
| Large Intestine, NOS | 1 | 0.13 | 98.44 |
| Mesothelioma | 1 | 0.13 | 98.57 |
| Nose, Nasal Cavity and Middle Ear | 1 | 0.13 | 98.7 |
| Penis | 1 | 0.13 | 98.83 |
| Rectosigmoid Junction | 1 | 0.13 | 98.96 |
| Retroperitoneum | 1 | 0.13 | 99.09 |
| Small Intestine | 1 | 0.13 | 99.22 |
| Stomach | 1 | 0.13 | 99.35 |
| Thymus | 1 | 0.13 | 99.48 |
| Tongue | 1 | 0.13 | 99.61 |
| Tonsil | 1 | 0.13 | 99.74 |
| Transverse Colon | 1 | 0.13 | 99.87 |
| Vagina | 1 | 0.13 | 100 |
| **Total** | **770** | **100** | 100 |

| **Supplementary Table 5. Demographics and clinicopathological characteristics after PSM** | | | |
| --- | --- | --- | --- |
| **Characteristics** | **No. of Patients (%)** | | ***P*** |
|  | **Second primary TC (Group1)**  **n=4,004** | **Primary TC**  **(Group2)**  **n=4,004** |  |
| **Age at diagnosis:**  Mean ± SD, y | 61.45±12.90 | 61.45±12.90 | 1 |
| **Gender** |  |  | 1 |
| Female | 2,851(71.20) | 2,851(71.20) |  |
| Male | 1,153(28.80) | 1,153(28.80) |  |
| **Race** |  |  |  |
| White | 3,329(83.14) | 3,238(80.87) | 0.057 |
| Black | 191(4.77) | 197(4.92) |  |
| Other | 475(11.83) | 526(13.14) |  |
| Unknown | 9(0.22) | 43(1.07) |  |
| **Year of diagnosis** |  |  | 0.073 |
| 1975-1990 | 0(0) | 0(0) |  |
| 1991-2000 | 0(0) | 0(0) |  |
| 2001-2010 | 1,392(34.77) | 1,469(36.69) |  |
| 2011-2019 | 2,612(65.23) | 2,535(63.31) |  |
| **Tumor grade** |  |  | 1 |
| Grade I | 453(11.31) | 453(11.31) |  |
| Grade II | 38(0.95) | 38(0.95) |  |
| Grade III | 7(0.17) | 7(0.17) |  |
| Grade IV | 16(0.40) | 16(0.40) |  |
| Unknown | 3,490(87.16) | 3,490(87.16) |  |
| **Histologic type** |  |  | 1 |
| Papillary | 2,837(70.85) | 2,837(70.85) |  |
| Follicular | 143(3.57) | 143(3.57) |  |
| Papillary with follicular | 892(22.28) | 892(22.28) |  |
| Medullary | 38(0.95) | 38(0.95) |  |
| Oxyphilic | 63(1.57) | 63(1.57) |  |
| Other | 31(0.77) | 31(0.77) |  |
| **T stage** |  |  | 1 |
| T0 | 3(0.07) | 3(0.07) |  |
| T1 | 2,747(68.61) | 2,747(68.61) |  |
| T2 | 496(12.39) | 496(12.39) |  |
| T3 | 670(16.73) | 670(16.73) |  |
| T4 | 88(2.20) | 88(2.20) |  |
| **N stage** |  |  | 1 |
| N0 | 3,249(81.14) | 3,249(81.14) |  |
| N1 | 755(18.86) | 755(18.86) |  |
| **M stage** |  |  | 1 |
| M0 | 3,991(99.68) | 3,991(99.68) |  |
| M1 | 13(0.32) | 13(0.32) |  |
| **Surgery performed** |  |  | 1 |
| Yes | 3,883(96.98) | 3,883(96.98) |  |
| No/Unknown | 121(3.02) | 121(3.02) |  |
| **Chemotherapy** |  |  | 1 |
| Yes | 6(0.15) | 6(0.15) |  |
| No/Unknown | 3,998(99.85) | 3,998(99.85) |  |
| **Radiotherapy** |  |  | 1 |
| Yes | 1,490(37.21) | 1,490(37.21) |  |
| No/Unknown | 2,514(62.79) | 2,514(62.79) |  |
| **Died of TC** |  |  | 0.328 |
| Yes | 58(1.45) | 58(1.45) |  |
| No/Unknown | 3,946(98.55) | 3,946(98.55) |  |
| **Abbreviation**: TC, thyroid cancer | | | |
